# Low socio-economic groups are not overweight in India

**Published:** 2011-01

**Authors:** Malavika A. Subramanyam, S.V. Subramanian

**Affiliations:** Center for Integrative Approaches to Health Disparities, School of Public Health, University of Michigan, MI, USA; * Department of Society, Human Development and Health, Harvard School of Public Health, Boston, MA, USA

Sir,

We commend Jeemon and Reddy^1^ for their review article focusing on the need to consider a social determinants perspective for understanding and addressing chronic diseases, including their risk factors such as overweight/obesity, in India. Here we highlight an inaccuracy in their specific claim that “obesity or overweight and hypertension are now associated with lower levels of education and income in India”^1^. The study cited by them does not appear to substantiate their claim with regards to overweight/obesity^2^, and shows that between 1995 and 2002 the increase in obesity and truncal obesity was substantially greater among individuals with higher levels of education. Another study by Reddy *et al*^3^again shows a clear positive association between overweight and education among men and women. While overweight appeared to be inversely associated with education in “urban” areas,^3^ the sample used in this study was solely based on employees working in large industries and their families^4^, representing a very small minority of the Indian population. On the other hand, studies using nationally representative data on adults from India, show unequivocally that overweight is concentrated primarily among high socio-economic groups (Fig. 1)^5^,^6^. Indeed, a positive association between overweight and socio-economic status (SES) has also been replicated in more than 50 developing countries^7^. We are intrigued, and concerned, with regards to the discordance between the data and interpretation that has also has been prevalent in other studies on obesity in developing countries^8^.

**Fig.**
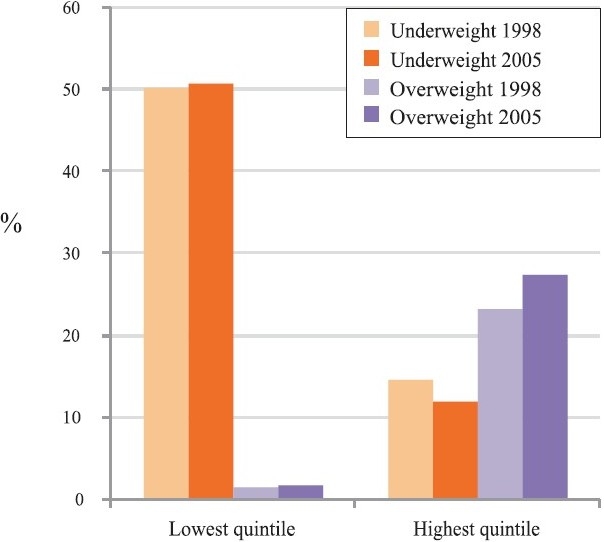
Per cent underweight (BMI<18.5 kg/m2) and overweight (BMI ≥25 kg/m2) in 1998-1999 and 2005-2006 among adult women in the lowest and highest quintile of household wealth from the 1998-1999 and 2005-2006 Indian National Family Health surveys (*Source*: Adapted from Ref. 5).

The idea that the association between SES and weight is initially positive and then flips to be being negative is seemingly drawn from experiences of rich, industrialized societies, even though our literature search did not yield a single empirical study that demonstrates this convincingly. Factors such as cheap availability of calorie-dense food, and food in general, dramatic shifts in occupational patterns from an agrarian to a service economy, high SES groups cutting down or shifting their dietary patterns, or economic growth spilling over to the low SES groups and improving their incomes in a substantial manner have to be occurring in order to anticipate a reversal in the SES-weight association. To our knowledge, there is little evidence of such changes occurring in India. On the contrary, inflation in food commodities has become an important concern^9^, economic growth has been extremely uneven and concentrated in a small minority^10^, and according to the 2001 Indian Census more than 70 per cent of the population continues to live in rural areas and more than half of the workforce remains engaged in labour-intensive agriculture activity^11^.

While increasing overweight prevalence is an important public health concern in India, it is premature and erroneous to characterize this as an emerging problem among the low SES groups in India.
